# Prognostic Implications of ALDH1 and Notch1 in Different Subtypes of Oral Cancer

**DOI:** 10.1155/2021/6663720

**Published:** 2021-02-13

**Authors:** Silas Antonio Juvencio de Freitas Filho, Cláudia Malheiros Coutinho-Camillo, Katia Klug Oliveira, Bárbara Beltrame Bettim, Clóvis Antônio Lopes Pinto, Luiz Paulo Kowalski, Denise Tostes Oliveira

**Affiliations:** ^1^Department of Surgery, Stomatology, Pathology and Radiology (Area of Pathology), Bauru School of Dentistry, University of São Paulo, Bauru, São Paulo, Brazil; ^2^International Research Center, A.C.Camargo Cancer Center, São Paulo, Brazil; ^3^Department of Pathology, A.C.Camargo Cancer Center, São Paulo, Brazil; ^4^Department of Head and Neck Surgery and Otorhinolaryngology, A.C.Camargo Cancer Center, São Paulo, Brazil

## Abstract

**Background:**

The present study aimed to investigate the clinical significance and prognostic value of the immunoexpression of cancer stem cell markers, ALDH1 and Notch1, in subtypes of oral squamous cell carcinoma.

**Materials and Methods:**

The expression of ALDH1 and Notch1 in 63 patients with well and poorly differentiated oral squamous cell carcinomas and their subtypes, verrucous carcinoma and basaloid squamous cell carcinoma, was evaluated by immunohistochemistry. The semi-quantitative analysis of the ALDH1 and Notch immunoexpression levels, based on the capture of 10 microscopic fields, at 400X magnification, at the invasive tumor front was performed and associated with clinicopathological variables using the chi-square test or Fisher's exact test. The overall and disease-free survival rates were estimated according to the Kaplan–Meier method and the curves were compared using the log-rank test. The independent effects of variables were calculated using Cox's proportional hazards regression model.

**Results:**

Strong ALDH1 and Notch1 expression was observed in 16 (25.4%) and 27 (42.9%) oral squamous cell carcinomas including their subtypes, respectively. Most tumors with strong immunoexpression of ALDH1 were basaloid squamous cell carcinoma (56.3%). Statistically significant associations were observed between the strong immunoexpression of Notch1 in poorly differentiated oral squamous cell carcinoma with perineural infiltration (*p* = 0.011) and lymph node involvement (pN+) (*p* = 0.034). The strong immunoexpression of ALDH1 was a prognostic factor associated with worse overall survival (*p* = 0.040) for patients with oral cancer.

**Conclusion:**

The strong immunoexpression of Notch1 can contribute to identification of patients with poorly differentiated oral squamous cell carcinoma, who have perineural infiltration or lymph node metastasis. In addition, the strong immunoexpression of ALDH1 may help to identify a worse prognosis in patients with oral squamous cell carcinoma and their subtypes.

## 1. Background

Cancer stem cells (CSCs), a subpopulation of cells with the capacity for maintaining tumor self-renewal, promoting recurrence and metastasis, have been identified through the expression of several cellular markers such as ALDH1 and Notch1 [1, 2]. Investigations to explore the use of CSC as therapeutic targets or prognostic indicators for patients, and their identification through biomarkers, have become the focus of several studies, including in head and neck squamous cell carcinoma [1, 3–5].

The ALDH1, an isoform 1 of the enzyme aldehyde dehydrogenase, is responsible for the oxidation of the aldehyde to carboxylic acid and performs the hydrolysis of esters, plays an antioxidant role, and serves as a binding protein for other molecules [6]. Its participation in the conversion of retinol to retinoic acid promotes the transcription of genes that participate in cell differentiation, apoptosis, or even tumor growth [6, 7].

Particularly in oral cancer, the clinical significance and prognostic value of ALDH1 immunoexpression by tumor cells have revealed a significant association with lymph node metastasis, histopathological tumor classification, local recurrence, and tumor location and with worse prognosis for patients with this malignancy [8–15]. However, many other investigations have failed to find significant association of the expression of ALDH1 with clinicopathological characteristics or with the prognosis of patients with oral cancer [4, 16].

The Notch1, a protein of the Notch signaling pathway, plays a fundamental role in several cellular processes such as proliferation and angiogenesis and in the epithelial-mesenchymal transition process, and its expression is well established in oral squamous cell carcinoma (OSCC) [17, 18]. Immunohistochemical analyses of Notch1 in OSCCs showed that its positivity in tumor cells was significantly associated with advanced clinical staging, lymph node metastasis, histopathological tumor classification, tumor invasion, and locoregional recurrence [18–24]. However, relative to the prognosis of patients with oral cancer, only one study showed that Notch1 immunoexpression was associated with worse overall survival [18].

Despite the various studies involving CSC markers such as ALDH1 and Notch1 in OSCC, the immunoexpression levels of these biomarkers specifically in subtypes of oral cancer with distinct clinical and prognostic behaviors such as verrucous carcinoma and basaloid squamous cell carcinoma are scarce in the scientific literature. Therefore, the aim the present study was to investigate the clinical value and prognostic implications of ALDH1 and Notch1 immunoexpression in subtypes of OSCC.

## 2. Materials and Methods

### 2.1. Patients and Tumor Samples

This study was based on the analysis of 63 surgical specimens from patients with primary OSCC treated at the Department of Head and Neck Surgery and Otorhinolaryngology of the A.C.Camargo Cancer Center, São Paulo, Brazil (1970–2013). The inclusion criteria were (1) patients with the diagnosis of well or poorly differentiated OSCC or basaloid squamous cell carcinoma (BSCC) or verrucous carcinoma, located in the oral cavity, confirmed by histopathological analysis; (2) patients initially submitted to surgical treatment, with or without adjuvant radiotherapy and/or chemotherapy; (3) patients with complete clinical follow-up; and (4) availability of paraffin blocks with representative fragments of the respective tumors for immunohistochemistry analyses.

The histopathological classification of the OSCCs was determined after microscopic analysis in H&E stained sections, according to the present criteria of the World Health Organization [25]. Demographic and clinical data (gender, age, smoking, drinking, tumor location, TNM stage-UICC/2004, treatment, and follow-up) were collected from the medical records of the A.C.Camargo Cancer Center. Pathological data related to angiolymphatic invasion, perineural infiltration, muscular infiltration, bone infiltration, and lymph node status (pN) were obtained from anatomopathological reports.

This study was approved by the Research Ethics Committee of the Bauru School of Dentistry, University of São Paulo (CAAE #97404918.0.0000.5417).

### 2.2. Immunohistochemistry

The immunohistochemistry technique of the present study followed the protocol of the International Research Center of the A.C.Camargo Cancer Center. Briefly, the 3 *μ*m tissue sections were dewaxed and dehydrated. For the ALDH1 antibody, antigen retrieval was performed with citrate solution (10 mM, pH = 6.0) and for Notch1 antibody, the EDTA solution (10 m, pH = 8.0) was used, both at 110°C, in a pressurized chamber for 15 minutes. The endogenous peroxidase and nonspecific protein activity were blocked with 3% aqueous hydrogen peroxide and Protein Block Serum-Free (Dako, Carpinteria, CA, USA), respectively, followed by incubation with the primary antibodies in a humid chamber (4°C-18 hours): anti-ALDH1 antibody (clone 44, BD Biosciences, San Jose, CA, USA, 1 : 500) and anti-Notch1 antibody (clone D1E11, Cell Signaling Technology, Beverly, MA, USA, 1 : 200). After revelation with adequate chromogenic substrate reaction of 3,3′-diaminobenzidine tetrahydrochloride (DAB, Dako, Carpinteria, CA, USA), the sections were stained with Harris Hematoxylin.

### 2.3. Immunohistochemistry Evaluation

In order to evaluate the immunoexpression of ALDH1 and Notch1, a total of ten microscopic fields at the invasive tumor front were captured sequentially, with a high-resolution digital camera (Axiocam MRc, Zeiss, Jena, Germany) coupled to the binocular optical microscope (Axioskop 2 Plus, Zeiss, Jena, Germany) with a 40x objective.

The immunoexpression levels of ALDH1 and Notch1 were evaluated by two pathologists, previously calibrated according to the semi-quantitative score system [26]. The score for each microscopic field consisted of the sum of the immunostaining intensity values: 0 (negative), 1 (weak), 2 (moderate), 3 (strong), or 4 (very strong); with the percentage of positive neoplastic cells: 0 (negative), 1 (<25%), 2 (25–50%), 3 (50–75%), or 4 (>75%). The tumors were finally classified as follows: 0 (score 0): absent immunopositivity staining; 1 (scores from 1 to 5): weak immunopositivity staining; 2 (scores from 6 to 8): strong immunopositivity staining.

### 2.4. Statistical Analysis

The association of the immunoexpression of ALDH1 and Notch1 with clinicopathological variables was analyzed using the chi-square (*x*^2^) or Fisher's exact test. The probabilities of overall and disease-free survivals, in five years, were estimated using the Kaplan–Meier method and compared by the log-rank test. The relative risk and independent effects of the significant pathological variables were evaluated using the univariate Cox proportional hazards model. For these statistical tests, tumors with negative and weak immunoexpression of ALDH1 or Notch1 were grouped, obtaining two groups: negative/weak immunopositivity staining and strong immunopositivity staining. For survival analysis, we dichotomized the well-differentiated OSCCs and verrucous carcinomas into a group of well-differentiated oral tumors, and the poorly differentiated OSCCs and BSCCs into a group of poorly differentiated oral tumors. For all statistical analyses, *p* values lower than 0.05 were considered statistically significant. Statistical analysis was performed using SPSS software-version 25.0 (SPSS Inc., Chicago, IL, USA).

## 3. Results

### 3.1. Patients and Tumor Samples

The majority of patients were male (68.3%), with a mean age of 60 years, smokers (84.2%), alcoholics (78.9%), with tumors located on the tongue or floor of the mouth (46.0%). In addition, most tumors were classified as T advanced clinical stage (58.8%), the clinical lymph node involvement was observed in 29 patients (46.0%), and ipsilateral cervical lymph node resection was performed in 31 patients (49.2%).

Of the 63 tumors, 20 were classified as well-differentiated OSCCs, 17 as poorly differentiated OSCCs, seven as verrucous carcinomas, and 19 as BSCCs. Angiolymphatic invasion was found in 21.3% of the tumors, and the majority were BSCC (53.8%). Perineural infiltration, muscle infiltration, and bone infiltration were observed in 40.0%, in 63.6%, and in 22.6% of the OSCCs, respectively. Histopathological lymph node metastasis (pN+) was detected in 53.2% of oral cancer patients. Locoregional recurrences occurred in 47.6% of those patients with OSCCs.

### 3.2. Immunoexpression for ALDH1 and Notch1

**Figure 1:**
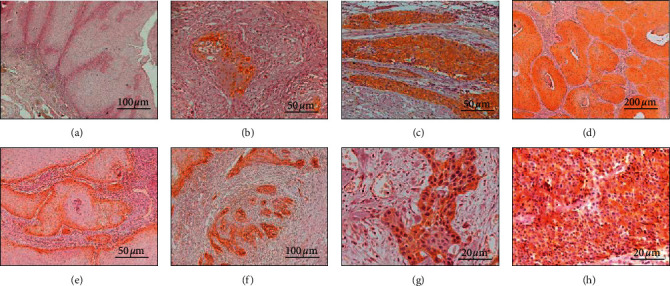
Immunohistochemistry for ALDH1 (a-d) and Notch1 (e-h) in subtypes of oral squamous cell carcinomas. The ALDH1 showed negative immunoexpression in the verrucous carcinoma (a) and the strong cytoplasmic immunoexpression of ALDH1 was observed in well-differentiated (b), in poorly differentiated (c), and in basaloid (d) oral squamous cell carcinomas. The membranous immunopositivity of Notch1 is illustrated in peripheral cells of the verrucous carcinoma (e) and the Notch1 cytoplasmic expression can be observed in well-differentiated (f), in poorly differentiated (g), and in basaloid (h) squamous cell carcinomas (immunohistochemistry: anti-ALDH1–a = 100*x*, b-c = 200*x*, *d* = 50*x*; anti-Notch1–*e* = 200*x*, *f* = 100*x*, g-h = 400*x*).

The ALDH1 was expressed mainly in the cytoplasm of neoplastic epithelial cells, while Notch1 exhibited cytoplasmic and/or membranous immunoexpression in OSCCs (Figure 1).

Table 1 shows that strong immunohistochemical expression for ALDH1 was observed in 16 of the 63 tumors (25.4%) with significant difference among the subtypes of oral cancer (*p* = 0.021), and the majority were BSCCs (56.3%). In addition, strong immunoexpression for Notch1 was observed in 27 of the 63 tumors (42.9%), the majority of which were well-differentiated OSCCs (40.7%).

Statistical analysis showed that there was no significant association between the expression of ALDH1 and the clinicopathological variables of patients with subtypes of OSCCs (Table 2).

Immunoexpression of Notch1 in poorly differentiated OSCCs was significantly associated with perineural infiltration (*p* = 0.011) and with pN+ (*p* = 0.034), as described in Table 3.

No association of ALDH1 and Notch1 with clinicopathological variables was found in the verrucous carcinomas due to the reduced number of tumor samples (*n* = 7). All verrucous carcinomas showed negative/weak ALDH1 immunoexpression, and strong Notch1 immunopositivity was observed in three tumors (42.9%).

### 3.3. Survival Analysis

The clinicopathological variables that showed statistically significant differences in the overall survival of patients were location of the primary tumor, T stage, N stage, histopathological tumor classification, radiotherapy, pN, and ALDH1 immunoexpression. Relative to disease-free survival, the clinicopathological variables with a statistically significant difference were N stage, tumor histopathological classification, radiotherapy, and pN. These results are described in Table 4.

**Figure 2:**
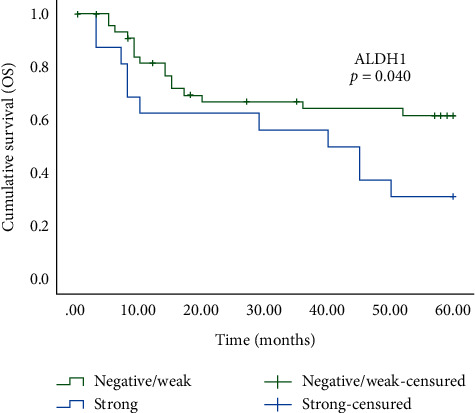
Overall survival (OS) analysis curves in patients with subtypes of oral cancer showing statistically significant differences between negative/weak (green line) and strong (blue line) ALDH1 immunoexpression. The *p* value was determined by log-rank test. *p* < 0.05 was considered statistically significant.

Figure 2 shows the comparison between the overall survival rate curves considering ALDH1 immunoexpression (*p* = 0.040) of the 63 patients with oral cancer. The patients with strong ALDH1 immunoexpression at the invasive tumor front had a lower overall survival in five years, compared with patients with negative/weak immunoexpression of this protein.

The univariate Cox proportional regression analysis revealed that patients with poorly differentiated oral tumors (poorly differentiated OSCCs and BSCCs) and with strong immunoexpression of ALDH1 had a higher risk of death (OR = 4.546, *p* = 0.002; OR = 2.176, *p* = 0.047, respectively). However, ALDH1 immunoexpression has failed to maintain its prognostic role in Cox's multivariate overall survival analysis (*p* > 0.05). In addition, as regards disease-free survival, the main variables indicating a worse prognosis for patients with oral cancer were poorly differentiated oral tumors (*p* = 0.007) and pN+ (*p* = 0.032). These results are shown in Table 5.

## 4. Discussion

The identification and quantification of CSCs have contributed to a better understanding of the biological behavior of different types of cancer, including oral cancer. To improve the understanding of the clinical and biological behavior of oral cancer and its subtypes, such as verrucous carcinoma and BSCC, aiming at the validation of CSC markers as prognostic indicators, the present investigation was carried out.

Previous studies regarding the immunoexpression of CSC markers, ALDH1 and Notch1, have investigated the associations with clinicopathological variables and/or with the prognosis of patients with oral cancer according to the immunostaining pattern of expression. However, there is still no consensus and many results have shown wide variation in the influence of these markers on the prognosis of patients with oral cancer. In addition, these studies concentrated on the analysis of these markers in conventional OSCCs. This study added contributions to the knowledge of immunoexpression and the prognostic value of CSC markers in the histological subtypes of OSCC, particularly the verrucous carcinoma and the BSCC.

In the present study, strong immunoexpression of ALDH1 was observed in only 16 of the 63 (25.4%) tumors, reinforcing the results obtained in other studies [4, 14, 16]. However, other research data have shown that strong immunoexpression of ALDH1 has been found in over 50% of neoplastic cells in oral cancer [9, 11, 13, 15]. Strong immunoexpression of Notch1 was observed in 27 of the 63 oral tumors (42.9%), a result that corroborated the findings of Upadhyay et al. [22]. However, the majority of studies have shown that over 50% of OSCCs expressed moderate or strong positivity for Notch1 [18–21, 23, 24, 27]. The difference in results probably occurred due to the different methods used for evaluating the immunoexpression.

We observed a statistically significant association between ALDH1 immunoexpression and the histologic grade of tumors (*p* = 0.021), revealing that most tumors with strong immunoexpression of this marker were classified as BSCCs (56.3%). Other studies have also found a statistically significant association between the strong immunoexpression of ALDH1 and the histologic grade, showing that this marking pattern occurs mainly in moderately or poorly differentiated OSCCs [8, 14, 15]. Our results reinforce the hypothesis that a higher density of CSCs in oral cancer is closely associated with a lower degree of cell differentiation, as also seen by Michifuri et al. and Tamatani et al. [8, 14].

The statistical comparison between four subtypes of oral cancer relative to the immunoexpression of Notch1 showed no significant difference (*p* = 0.555; Table 1), a result supported by several other studies [18, 20, 23, 28], whereas Gan et al. and Ravindran and Devaraj observed a statistically significant difference when comparing the immunoexpression of Notch1 in OSCCs with different degrees of cell differentiation [24, 27]. The results of these studies, including ours, are controversial and need to be confirmed in further studies for better understanding of the role of this protein in tumor biology.

There was no association between the immunoexpression of ALDH1 and the clinicopathological variables of patients with different histopathological subtypes of OSCCs, a result very similar to that obtained by de Moraes et al. and Wu et al. [4, 10]. However, many studies with this marker have shown significant associations, mainly between the strong immunoexpression of ALDH1 and lymph node metastasis [8, 11, 13, 14] and with the advanced clinical stage [13, 15] of patients with oral cancer.

For Notch1, statistically significant associations were observed between its strong immunohistochemical expression in neoplastic cells and perineural infiltration (*p* = 0.011) and with lymph node metastasis (*p* = 0.034) of patients with poorly differentiated OSCCs (Table 3). Some authors have described an association of the strong immunoexpression of Notch1 with advanced clinical stage and/or with lymph node metastasis in patients with oral cancer [18–24]. Ravindran and Devaraj observed a statistically significant association of weak or negative immunoexpression of Notch1 with advanced clinical stage and with lymph node metastasis of patients with oral cancer, suggesting that the absence of immunoexpression of this marker could be related to the processes of invasion and metastasis [27].

The analysis of survival probabilities for the 63 patients with oral cancer in 5 years showed a lower probability of overall survival, with a significant difference, for patients with strong immunoexpression of ALDH1 (*p* = 0.040; Table 4 and Figure 2). In addition, the univariate analysis using the Cox model showed that patients with oral cancer and strong ALDH1 immunoexpression had a 2.176 times higher relative risk of death when compared with patients with negative or weak immunoexpression of this marker (*p* = 0.047; Table 5). Our results contrast with others in which ALDH1 immunoexpression had no prognostic value in the overall survival of patients with oral cancer [10, 13, 16]. However, Wang et al. demonstrated an association of the strong ALDH1 immunoexpression with worse overall survival, which was an independent prognostic factor for patients with oral cancer [15]. Contrary to the results of Tamatani et al. [14], our study found no significant association between the ALDH1 immunoexpression and disease-free survival in patients with oral cancer.

In the present study, we observed that the immunoexpression of Notch1 did not influence the prognosis of patients with oral cancer (Table 4), a result similar to those obtained by Joo et al. and Ravindran and Devaraj [19, 27]. Contrasting with these results, Wang et al. found that positivity for Notch1 indicated lower overall survival and was an independent prognostic factor for patients with oral cancer, but in the cited study only five tumor regions were selected for immunohistochemistry analysis [18].

Our study showed that CSC markers, ALDH1 and Notch1, were infrequently found in verrucous carcinoma, a result that could be justified by the indolent behavior of this tumor, whereas the strong immunopositivity of ALDH1 in poorly differentiated oral tumors, especially in the BSCCs, may indicate a higher degree of aggressiveness in the clinical behavior of these subtypes of oral cancer. Moreover, strong immunoexpression of Notch1 was useful in cases of some clinicopathological variables of patients with poorly differentiated OSCCs, once again revealing their participation in oral neoplasms according to the pattern of tumor cell differentiation.

The univariate Cox proportional regression analysis showed that patients with poorly differentiated oral tumors have a significantly higher relative risk of death and risk of locoregional recurrence when compared with patients with well-differentiated oral tumors (Table 5). Regarding BSCC, previous studies have shown that its clinical behavior and the rates of overall and disease-free survival are similar to those of poorly differentiated OSCC, and the same treatment protocols can be applied [29–31]. Probably the factor that had the most influence on the prognosis of patients with oral cancer, which was also observed in this study, is lymph node metastasis (pN+) [15, 18, 32]. The present study showed a significantly increased risk for locoregional recurrence (Table 5).

## 5. Conclusion

In summary, strong immunoexpression of Notch1 can contribute to the identification of patients with poorly differentiated OSCCs who have perineural infiltration and lymph node metastasis. Moreover, strong immunoexpression of ALDH1 may help to predict a worse prognosis in the overall survival of patients with oral cancer.

## Figures and Tables

**Table 1:** Distribution of the frequency of ALDH1 and Notch1 immunoexpression in the 63 patients with subtypes of oral cancer.

Antibodies and immunoexpression	Subtypes of oral cancer					*p*
	WDOSCC *N* (%)	PDOSCC *N* (%)	VC *N* (%)	BSCC *N* (%)	Total *N* (%)	
ALDH1						
Negative/weak	18 (38.3)	12 (25.5)	7 (14.9)	10 (21.3)	47 (100)	**0.021**
Strong	2 (12.4)	5 (31.3)	0 (0)	9 (56.3)	16 (100)	

Notch1						
Negative/weak	9 (25.0)	10 (27.8)	4 (11.1)	13 (36.1)	36 (100)	0.555
Strong	11 (40.7)	7 (26.0)	3 (11.1)	6 (22.2)	27 (100)	

WDOSCC, well-differentiated oral squamous cell carcinoma; PDOSCC, poorly differentiated squamous cell *carcinoma*; VC, verrucous carcinoma; BSCC, basaloid squamous cell carcinoma; *N*, number of tumors. Analysis by chi-square test. *p* < 0.05 was considered statistically significant.

**Table 2:** Association between clinicopathological variables and ALDH1 immunoexpression in patients with subtypes of oral cancer.

Variable	ALDH1 immunoexpression in subtypes of oral cancer								
	WDOSCC		*p*	PDOSCC		*p*	BSCC		*p*
	(−)	(+)		(−)	(+)		(−)	(+)	
	*N* (%)	*N* (%)		*N* (%)	*N* (%)		*N* (%)	*N* (%)	
Gender									
Male	9 (50.0)	2 (100)	0.479	9 (75.0)	5 (100)	0.515	9 (90.0)	7 (77.3)	0.582
Female	9 (50.0)	0		3 (25.0)	0		1 (10.0)	2 (22.2)	

Age									
≤60 years	8 (44.4)	2 (100)	0.474	7 (58.3)	4 (80.0)	0.600	4 (40.0)	2 (22.2)	0.628
>60 years	10 (55.8)	0		5 (41.7)	1 (20.0)		6 (60.0)	7 (77.8)	

Tobacco^*∗*^									
Yes	6 (75.0)	2 (100)	1.000	6 (85.7)	5 (100)	1.000	6 (85.7)	6 (100)	1.000
No	2 (25.0)	0		1 (14.3)	0		1 (14.3)	0	

Alcohol^*∗*^									
Yes	5 (55.6)	2 (100)	0.491	6 (100)	5 (100)	NC	4 (66.7)	5 (83.3)	1.000
No	4 (44.4)	0		0	0		2 (33.8)	1 (16.7)	

T stage									
T1-T2	10 (55.6)	1 (50.0)	1.000	1 (8.3)	1 (20.0)	0.515	4 (40.0)	2 (22.2)	0.628
T3-T4	8 (44.4)	1 (50.0)		11 (91.7)	4 (80.0)		6 (60.0)	7 (77.8)	

N stage									
N0	14 (77.8)	2 (100)	1.000	4 (33.3)	1 (20.0)	1.000	4 (44.4)	1 (11.1)	0.294
N+	4 (22.2)	0		8 (66.7)	4 (80.0)		5 (55.6)	8 (88.9)	

Radiotherapy									
Yes	4 (22.2)	2 (100)	0.079	10 (83.3)	4 (80.0)	1.000	6 (60.0)	8 (88.9)	0.303
No	14 (77.8)	0		2 (16.7)	1 (20.0)		4 (40.0)	1 (11.1)	

Recurrence									
Yes	7 (38.9)	0	0.521	5 (41.7)	3 (60.0)	0.620	7 (70.0)	6 (66.7)	1.000
No	11 (61.1)	2 (100)		7 (58.3)	2 (40.0)		3 (30.0)	3 (33.3)	

Angiolymphatic invasion^*∗*^									
Yes	3 (17.6)	0	1.000	2 (18.2)	1 (20.0)	1.000	3 (30.0)	4 (44.4)	0.650
No	14 (82.5)	2 (100)		9 (81.8)	4 (80.0)		7 (70.0)	5 (55.6)	

Perineural infiltration^*∗*^									
Yes	8 (47.1)	1 (50.0)	1.000	8 (72.7)	2 (40.0)	0.299	4 (44.4)	1 (11.1)	0.294
No	9 (52.9)	1 (50.0)		3 (27.3)	3 (60.0)		5 (55.6)	8 (88.9)	

Lymph node (pN)^*∗*^									
Yes	3 (25.0)	0	1.000	8 (66.7)	3 (75.0)	1.000	5 (62.5)	6 (66.7)	1.000
No	9 (75.0)	2 (100.0)		4 (33.3)	1 (25.0)		3 (37.5)	3 (33.3)	

WDOSCC, well-differentiated oral squamous cell carcinoma; PDOSCC, poorly differentiated squamous cell carcinoma; BSCC, basaloid squamous cell carcinoma; N, number of tumors; NC, not calculated. Analysis by chi-square test or Fischer's exact test. *p* < 0.05 was considered statistically significant. ^*∗*^Excluded patients without records.

**Table 3:** Association between clinicopathological variables and Notch1 immunoexpression in patients with subtypes of oral cancer.

Variable	Notch1 immunoexpression in subtypes of oral cancer								
	WDOSCC		*p*	PDOSCC		*p*	BSCC		*p*
	(−)	(+)		(−)	(+)		(−)	(+)	
	*N* (%)	*N* (%)		*N* (%)	*N* (%)		*N* (%)	*N* (%)	
Gender									
Male	7 (77.8)	4 (36.4)	0.092	7 (70.0)	7 (100)	0.228	11 (84.6)	5 (83.3)	1.000
Female	2 (22.2)	7 (63.6)		3 (30.0)	0		2 (15.4)	1 (16.7)	

Age									
≤60 years	5 (55.6)	4 (36.4)	1.000	7 (70.0)	4 (57.1)	0.644	4 (30.8)	2 (33.3)	1.000
>60 years	4 (44.4)	7 (63.3)		3 (30.0)	3 (42.9)		9 (69.2)	4 (66.7)	

Tobacco^*∗*^									
Yes	2 (100)	6 (75.0)	1.000	7 (87.5)	4 (100)	1.000	8 (88.9)	4 (100)	1.000
No	0	2 (25.0)		1 (12.5)	0		1 (11.1)	0	

Alcohol^*∗*^									
Yes	3 (100)	4 (50.0)	0.236	7 (100)	4 (100)	NC	7 (87.5)	2 (50.0)	0.236
No	0	4 (50.0)		—	—		1 (12.5)	2 (50.0)	

T stage									
T1-T2	6 (66.7)	5 (45.5)	0.406	2 (20.0)	0	0.485	5 (38.5)	1 (16.7)	0.605
T3-T4	3 (33.3)	6 (54.5)		8 (80.0)	7 (100)		8 (61.5)	5 (83.3)	

N stage									
N0	8 (88.9)	8 (72.7)	0.591	4 (40.0)	1 (14.3)	0.338	2 (16.7)	3 (50.0)	0.268
N+	1 (11.1)	3 (27.3)		6 (60.0)	6 (85.7)		10 (83.3)	3 (50.0)	

Radiotherapy									
Yes	3 (33.3)	3 (27.3)	1.000	8 (80.0)	6 (85.7)	1.000	10 (76.9)	4 (66.7)	1.000
No	6 (66.7)	8 (72.7)		2 (20.0)	1 (14.3)		3 (23.1)	2 (33.3)	

Recurrence									
Yes	5 (55.6)	2 (18.2)	0.160	4 (40.0)	4 (57.1)	0.637	10 (76.9)	3 (50.0)	0.320
No	4 (44.4)	9 (81.8)		6 (60.0)	3 (42.9)		3 (23.1)	3 (50.0)	

Angiolymphatic invasion^*∗*^									
Yes	1 (12.5)	2 (18.2)	1.000	1 (11.1)	2 (28.6)	0.550	5 (38.5)	2 (33.3)	1.000
No	7 (87.5)	9 (81.8)		8 (88.9)	5 (71.4)		8 (61.5)	4 (66.7)	

Perineural infiltration^*∗*^									
Yes	5 (62.5)	4 (36.4)	0.370	3 (33.3)	7 (100)	**0.011**	4 (33.3)	1 (16.7)	0.615
No	3 (37.5)	7 (63.6)		6 (66.7)	0		8 (66.7)	5 (83.3)	

Lymph node (pN)^*∗*^									
Yes	1 (20.0)	2 (22.2)	1.000	4 (44.4)	7 (100)	**0.034**	8 (66.7)	3 (60.0)	1.000
No	4 (80.0)	7 (77.8)		5 (55.6)	0		4 (33.3)	2 (40.0)	

WDOSCC, well-differentiated oral squamous cell carcinoma; PDOSCC, poorly differentiated squamous cell carcinoma; BSCC, basaloid squamous cell carcinoma; N, number of tumors; NC, not calculated. Analysis by chi-square test or Fischer's exact test. *p* < 0.05 was considered statistically significant. ^*∗*^Excluded patients without records.

**Table 4:** Overall survival (OS) and disease-free survival (DFS) rates in 5 years of 63 patients with subtypes of oral cancer according to the clinicopathological variables, and ALDH1 and Notch1 immunoexpression.

Variable		Overall survival		Disease-free survival	
		5 years (%)	*p*	5 years (%)	*p*
Tobacco	No	66.7	0.407	83.3	0.228
	Yes	46.6		48.9	

Alcohol	No	75.0	0.298	62.5	0.901
	Yes	49.9		60.4	

Tumor site	Tongue/floor of mouth	40.6	**0.024**	54.1	0.716
	Palate	85.7		57.1	
	Retromolar/gingiva	40.6		49.2	
	Others	0		76.2	

T stage	T1-T2	75.5	**0.005**	65.2	0.276
	T3-T4	39.2		50.5	

N stage	N0	72.4	**0.001**	73.5	**0.005**
	N+	31.5		34.9	

Histologic grade	WDOSCC + VC	78.1	**0.001**	79.6	**0.004**
	PDOSCC + BSCC	34.7		37.6	

Radiotherapy	No	70.7	**0.028**	75.1	**0.037**
	Yes	39.6		42.0	

Perineural invasion	No	55.9	0.471	54.6	0.844
	Yes	44.9		55.3	

Lymph node (pN)	No	59.1	**0.045**	70.1	**0.024**
	Yes	32.6		34.7	

ALDH1	Negative/weak	61.7	**0.040**	59.6	0.458
	Strong	31.3		46.2	

Notch1	Negative/weak	49.9	0.810	48.0	0.110
	Strong	55.6		68.3	

WDOSCC, well-differentiated oral squamous cell carcinoma; VC, verrucous carcinoma; PDOSCC, poorly differentiated squamous cell carcinoma; BSCC, basaloid squamous cell carcinoma. *p* value obtained by log-rank test. *p* < 0.05 was considered statistically significant. The values highlighted in bold show that they were statistically significant.

**Table 5:** Univariate analysis for overall survival and disease-free survival in 63 patients with subtypes of oral cancer.

Variable	Overall survival		*p*	Disease-free survival		*p*
	HR	CI (95%)		HR	CI (95%)	
Histologic grade						
WDOSCC + VC(0)	4.546	[1.717; 12.036]	**0.002**	3.908	[1.446; 10.562]	**0.007**
PDOSCC + BSCC(1)						

Lymph node (pN)						
No (0)	2.250	[0.989; 5.115]	0.053	2.892	[1.094; 7.645]	**0.032**
Yes (1)						

ALDH1						—
Negative/weak (0)	2.176	[1.009; 4.696]	**0.047**	—	—	
Strong(1)						

WDOSCC, well-differentiated oral squamous cell carcinoma; VC, verrucous carcinoma; PDOSCC, poorly differentiated squamous cell carcinoma; BSCC, basaloid squamous cell carcinoma; HR, hazard ratio; CI, confidence interval; *p* value obtained by Cox test. *p* < 0.05 was considered statistically significant. The values highlighted in bold show that they were statistically significant.

## Data Availability

The data used to support the findings of this study are included within the article.
